# How to normalize Twitter counts? A first attempt based on journals in the Twitter Index

**DOI:** 10.1007/s11192-016-1893-6

**Published:** 2016-02-27

**Authors:** Lutz Bornmann, Robin Haunschild

**Affiliations:** Division for Science and Innovation Studies, Administrative Headquarters of the Max Planck Society, Hofgartenstr. 8, 80539 Munich, Germany; Max Planck Institute for Solid State Research, Heisenbergstr. 1, 70569 Stuttgart, Germany

**Keywords:** Twitter counts, Twitter percentiles, Twitter Index, Altmetrics

## Abstract

One possible way of measuring the broad impact of research (societal impact) quantitatively is the use of alternative metrics (altmetrics). An important source of altmetrics is Twitter, which is a popular microblogging service. In bibliometrics, it is standard to normalize citations for cross-field comparisons. This study deals with the normalization of Twitter counts (TC). The problem with Twitter data is that many papers receive zero tweets or only one tweet. In order to restrict the impact analysis on only those journals producing a considerable Twitter impact, we defined the Twitter Index (TI) containing journals with at least 80 % of the papers with at least 1 tweet each. For all papers in each TI journal, we calculated normalized Twitter percentiles (TP) which range from 0 (no impact) to 100 (highest impact). Thus, the highest impact accounts for the paper with the most tweets compared to the other papers in the journal. TP are proposed to be used for cross-field comparisons. We studied the field-independency of TP in comparison with TC. The results point out that the TP can validly be used particularly in biomedical and health sciences, life and earth sciences, mathematics and computer science, as well as physical sciences and engineering. In a first application of TP, we calculated percentiles for countries. The results show that Denmark, Finland, and Norway are the countries with the most tweeted papers (measured by TP).

## Introduction

The success of the modern science system is closely related to a functioning research evaluation system by peers: without critical assessments by peers improvements of research approaches would be absent and standards could not be reached (Bornmann 2011). With the advent of large bibliometric databases (especially the citation indexes of Thomson Reuters) and the need for cross-disciplinary comparisons (e.g. of complete universities) bibliometrics has been more and more used to supplement (or sometimes to replace) peer review. Various national research evaluation systems have a strong focus on bibliometrics (Bornmann in press) and a manifesto has been published how bibliometrics can be properly used in research evaluation (Hicks et al. 2015). Citation analyses measure the impact of science on science. Since governments are interested today not only in this recursive kind of impact, but also in the broad impact of science on the wider society, scientometricians are searching for new metrics measuring broad impact reliably and validly. The use of case studies for demonstrating broad impact in the current UK Research Excellence Framework (REF) is a qualitative approach with the typical problems of missing generalizability, great amount of work, and case selection bias (only favorable cases of impact are reported) (Bornmann 2013).

One possible way of measuring broad impact quantitatively is the use of alternative metrics (altmetrics) (Bornmann 2014a; NISO Alternative Assessment Metrics Project 2014)—a new subset of scientometrics (Priem 2014). “Alternative metrics, sometimes shortened to just altmetrics, is an umbrella term covering new ways of approaching, measuring and providing evidence for impact” (Adie 2014, p. 349). An important source of altmetrics is Twitter: It is a popular microblogging platform with several million active users and messages (tweets) being sent each day. Tweets are short messages which cannot exceed 140 characters in length (Shema et al. 2014). Direct or indirect links from a tweet to a publication are defined as Twitter mentions (Priem and Costello 2010). Twitter mentions can be counted (Twitter counts, TC) in a similar way as traditional citations and the impact of different publications can be compared.

In bibliometrics, it is standard to normalize citations (Wilsdon et al. 2015). Citations depend on publication year and subject category. Thus, for cross-field and cross-time comparisons normalized citation scores are necessary and have been developed in recent decades (Vinkler 2010). Against the backdrop of the general practice of normalizing citations, many authors in the area of altmetrics argue for the necessity to field- and time-normalize altmetrics, too (Fenner 2014; Taylor 2013). Recently, Haunschild and Bornmann (2016) have proposed methods to normalize Mendeley counts—a popular altmetrics based on data from an online reference manager. In this paper, we propose corresponding methods for TC so that Twitter impact can be fairly measured across papers published in different subject categories and publication years. Since Twitter data has other properties than Mendeley data, methods developed for Mendeley cannot simply be transferred to Twitter and new methods for Twitter data are in need.

## Research on Twitter

A free account on Twitter enables users to “follow” other Twitter users. This means one subscribes to their updates and can read their “tweets” (short messages) in a feed. Also, one can “retweet” these messages or tweet new short messages which are read by own followers in their feeds. Up to a third of the tweets may be simple retweets (Holmberg 2014). “Tweets and retweets are the core of the Twitter platform that allows for the large-scale and rapid communication of ideas in a social network” (Darling et al. 2013). Whereas at the start of the platform Twitter was mostly used for personal communication, studies have uncovered its increasing use for work-related purposes (Priem and Costello 2010; Priem and Hemminger 2010).

It is possible to include references to publications in tweets: “We defined Twitter citations as direct or indirect links from a tweet to a peer-reviewed scholarly article online” (Priem and Costello 2010). Since tweets are restricted to 140 characters, it is frequently difficult to explore why a paper has been tweeted (Haustein et al. 2014a). In most of the cases tweets including a reference to a paper will have the purpose of bringing a new paper to the attention of the followers. Thus, tweets are not used (and cannot be used) to extensively discuss papers. According to Haustein et al. (2014a) “unlike Mendeley, Twitter is widely used outside of academia and thus seems to be a particularly promising source of evidence of public interest in science” (p. 208). TC do not correlate with citation counts (Bornmann 2015) and the results of Bornmann (2014b) show that particularly well written scientific papers (not only understandable by experts in a field) which provides a good overview of a topic generate tweets.

The results of Haustein et al. (2014b) point to field differences in tweeting: “Twitter coverage at the discipline level is highest in Professional Fields, where 17.0 % of PubMed documents were mentioned on Twitter at least once, followed by Psychology (14.9 %) and Health (12.8 %). When the data set is limited to only those articles that have been tweeted at least once, the papers from Biomedical Research have the highest Twitter citation rate (T/P_tweeted_ = 3.3). Of the 284,764 research articles and reviews assigned to this discipline, 27,878 were mentioned on Twitter a total of 90,633 times. Twitter coverage is lowest for Physics papers covered by PubMed (1.8 %), and Mathematics papers related to biomedical research receive the lowest average number of tweets per tweeted document (T/P_tweeted_ = 1.5)” (p. 662). According to Zahedi, Costas, and Wouters (2014) “in Twitter, 7 % of the publications from Multidisciplinary field, 3 % of the publications from Social & Behavioural Sciences and 2 % of publications from Medical & Life Sciences are the top three fields that have at least one tweet. In Delicious, only 1 % of the publications from Multidisciplinary field, Language, Information & Communication and Social & Behavioural Sciences have at least one bookmark while other fields have less than 1 % altmetrics” (p. 1498).

The results of both studies indicate that TC should be normalized with respect to field assignments.

## Methods

### Data

We obtained the Twitter statistics for articles and reviews published in 2012 and having a DOI (*n*_A_ = 1,198,184 papers) from Altmetric^1^—a start-up providing article-level metrics—on May 11, 2015. The DOIs of the papers from 2012 were exported from the in-house database of the Max Planck Society (MPG) based on the Web of Science (WoS, Thomson Reuters) and administered by the Max Planck Digital Library (MPDL). We received altmetric data from Altmetric for 310,933 DOIs (26 %). Altmetric did not register altmetric activity for the remaining papers. For 37,692 DOIs (3 %), a Twitter count of 0 was registered. The DOIs with no altmetric activity registered by Altmetric were also treated as papers with 0 tweets. Furthermore, our in-house data base was updated in the meantime. 12,960 DOIs for papers published in 2012 were added (e.g. because new journals with back files were included in the WoS by Thomson Reuters). We treat these added papers as un-tweeted papers. Thus, a total of 937,843 papers (77 %) out of 1,211,144 papers were not tweeted.

### Normalization of Twitter counts

In the following, we propose a possible procedure for normalizing TC which is percentile-based. The procedure focusses on journals (normalization on the journal level) and pools the journals with the most Twitter activities in the so called Twitter Index (TI).

Following percentile definitions of Leydesdorff and Bornmann (2011), ImpactStory—a provider of altmetrics for publications—provides Twitter percentiles (TP) which are normalized according to the publication year and scientific discipline of papers (Chamberlain 2013; Roemer and Borchardt 2013). The procedure of ImpactStory for calculating the percentile for a given paper *i* is as follows^2^: (1) The discipline is searched at Mendeley (a citation management tool and social network for academics) from which paper *i* is most frequently read. “Saves” at Mendeley are interpreted in altmetrics as “reads” and Mendeley readers share their discipline. (2) All papers which are assigned to the same discipline in Mendeley and are published in the same year (these papers constitute the reference set of paper *i*) are sorted in descending order according to their TC. (3) The proportion of papers is determined in the reference set which received less tweets than paper *i*. (4) The proportion equals the percentile for paper *i*.

It is a sign of professional scientometrics to use normalized indicators. Compared to other methods used for normalization in bibliometrics, percentile-based indicators are being seen as robust indicators (Hicks et al. 2015; Wilsdon et al. 2015). However, the procedure used by ImpactStory has some disadvantages (as already outlined on its website): (1) There might be instances where a paper’s actual discipline doesn’t match the disciplinary reference set used for the normalization. Papers might be read in disciplines to which they do not belong. (2) The discipline for a paper might change, if the most frequently read discipline changes from one year to another. (3) If a paper does not have any readers at Mendeley, all papers within one year constitute the reference set in ImpactStory. It is clear that this change favors papers from certain disciplines then (e.g. life sciences). (4) The results of Haustein et al. (2014b) show that approximately 80 % of the articles published in 2012 do not receive any tweet. Most of the articles with tweets received only one tweet. The long tail of papers in the distribution of tweets with zero or only one tweet leads to high percentile values for papers, although they have only one or two tweets. (5) The results of Bornmann (2014b) show that many subject categories (in life sciences) are characterized by low average TC. Only very few categories show higher average counts. This is very different to mean citation rates which exhibit greater variations over the disciplines. The missing variation of average TC over the subject categories let Bornmann (2014b) come to the conclusion that TC should be normalized on a lower level than subject categories. The normalization on the journal level could be an alternative.

Against the backdrop of these considerations, we develop a first attempt to normalize TC properly in this study which improves the method used by ImpactStory. First of all, the normalization of TC only makes sense, if most of the papers in the reference sets have at least one tweet. Strotmann and Zhao (2015) published the 80/20 scientometric data quality rule: a reliable field-specific study is possible with a database, if 80 % of the field-specific publications are covered in this database. We would like to transfer this rule to Twitter data and propose to normalize Twitter data only then if the field-specific reference sets are covered with at least 80 % on Twitter (coverage means in this context that a publication has at least one tweet). We could use Mendeley disciplines (following ImpactStory) or—which is conventional in bibliometrics—WoS subject categories (sets of journals with similar disciplinary focus) for the normalization process. However, both solutions would lead to the exclusion of most of the fields (because they have more than 20 % papers with zero tweets). Thus, we would like to propose the normalization on the journal-level which is also frequently used in bibliometric studies (Vinkler 2010). Here, the reference set is constituted by the papers which are published in the same journal and publication year.

In this study, we use all articles and reviews published in 2012 as initial publication set. The application of the 80/20 scientometric data quality rule on the journals in the set leads to 413 journals with TC for at least 80 % of the papers (4.3 %) and 9242 journals with TC for less than 80 % (95.7 %). We propose to name the set of journals with high Twitter activity as TI. Because many TI journals have published only a low numbers of papers, we reduced the journals in the TI further on (this will be explained later on in this section). We propose to compose the TI every 12 months (e.g. by Twitter). In other words, every 12 months the journals should be selected in which at least 80 % of the papers had at least one tweet. Then, the papers in these journals are used for evaluative Twitter studies on research units (e.g. institutions or countries).

In order to normalize tweets, we propose to calculate percentiles (following ImpactStory) on the base of the tweets for every paper in a journal. There are several possibilities to calculate percentiles (it is not completely clear which possibility is used by ImpactStory). The formula derived by Hazen (1914) ((*i* − 0.5)/n*100) is used very frequently nowadays for the calculation of percentiles (Bornmann et al. 2013b). It is an advantage of this method that the mean percentile for the papers in a journal equals 50. Table 1 shows the calculation of TP for an example set of 11 publications in a journal. If the papers in a journal are sorted in descending order by their TC, *i* is the rank position of a paper and *n* is the total number of papers published in the journal. Paper no. 6 is assigned the percentile 50 because 50 % of the papers in the table have a higher rank (more tweets) and 50 % of the values have a lower rank (fewer tweets). Papers with equal TC are assigned the average rank *i* in the table. For example, as there are two papers with 44 tweets, they are assigned the rank 9.5 instead of the ranks 9 and 10.

**Table 1:** The calculation of Twitter percentiles for an example set of 11 publications in a journal

No.	Tweets	*i*	*n*	Twitter percentile
11	54	11	11	95.45
10	44	9.5	11	81.82
9	44	9.5	11	81.82
8	13	8	11	68.18
7	10	7	11	59.09
6	9	6	11	50.00
5	1	4	11	31.82
4	1	4	11	31.82
3	1	4	11	31.82
2	0	1.5	11	9.09
1	0	1.5	11	9.09

The TP are field-normalized impact scores. The normalization on the base of journals is on a lower aggregation level than the normalization on the basis of WoS subject categories (Bornmann 2014b). WoS subject categories are aggregated journals to journal sets. TP are proposed to use for comparisons between units in science (researchers, research groups, institutions, or countries) which have published in different fields.

## Results

The results which are presented in the section “Differences in Twitter counts between Twitter Index journals” show the differences in TC between the TI journals. We test the field-normalized Twitter scores in “Validation of Twitter percentiles using the fairness test” whether the field-normalization effectively works. In “Comparison of countries based on Twitter percentiles” , we present some results on the Twitter impact of countries which is field-normalized.

### Differences in Twitter counts between Twitter Index journals

For the calculation of the TP we have identified the 413 journals in 2012 with TC for at least 80 % of the papers. We further excluded 259 journals from the TI, because these journals had less than 100 papers published in 2012. For the calculation of the TP the paper set should not be too small and the threshold of 100 can be well justified: If all papers in a journal with at least 100 papers had different TC, all integer percentile ranks would be occupied. Thus, the set of journals in the TI is reduced from 413 journals with TC for at least 80 % of the papers to 156 journals which have also published at least 100 papers. Table 2 shows a selection of twenty journals with the largest average tweets per paper. A table with data for all 156 journals in the TI is located in the Appendix (see Table 6).

**Table 2:** Twenty journals in the Twitter Index with the largest average tweets per paper published in 2012

Journal	Number of papers	Sum of tweets	Average tweets	Median number of tweets	Percentage of tweeted papers
*New England Journal of Medicine*	215	16,908	78.6	53	100.0
*The Lancet*	233	10,750	46.1	21	100.0
*British Medical Journal*	325	12,469	38.4	20.5	98.8
*Journal of Medical Internet Research*	182	6398	35.2	19	98.9
*Psychological Science*	213	7024	33.0	11	96.7
*PLoS Medicine*	114	3754	32.9	17	100.0
*Science*	826	25,262	30.6	19	99.4
*Nature*	863	26,326	30.5	13	97.9
*Nature Climate Change*	120	3008	25.1	8	99.2
*Archives of Internal Medicine*	103	2491	24.2	4	97.1
*Science Translational Medicine*	211	4995	23.7	16	99.1
*American Journal of Clinical Nutrition*	325	6421	19.8	6	92.6
*Health Affairs*	268	5165	19.3	9	94.8
*Journal of the American Medical Association*	230	4350	18.9	8.5	98.7
*PLoS Biology*	152	2799	18.4	7	95.4
*Nature Medicine*	162	2862	17.7	11	96.9
*British Journal of Sports Medicine*	198	3329	16.8	5	89.4
*BMC Medicine*	126	2048	16.3	8	99.2
*Scientific Reports*	790	12,800	16.2	3	91.9
*British Dental Journal*	133	2129	16.0	12	100.0

The results in Tables 2 and 6 (in the Appendix) reveal a large heterogeneity between the journals with respect to the average and median number of tweets. Whereas the papers published in the *New England Journal of Medicine* have on average 78.6 tweets, the papers published in the *British Dental Journal* are tweeted on average 16 times. The large differences in average tweets already for the twenty most tweeted journals might demonstrate that the normalization of TC on the journal level seems sensible. In contrast to the results of Bornmann (2014b) on the level of subject categories (see the explanation of the study above), there is a greater variation of average tweets on the journal level. In other words, the TI journals are not characterized by only a few journals with very high average TC and most of the journals with low averages or nearly zero average TC. Thus, the level of journals seems proper for the normalization of TC.

Haustein et al. (2014a) found a broad interest by the general public in papers from the biomedical research, which is also reflected in the average TC in Tables 2 and 6: Many journals form the area of general biomedical research are among the most tweeted journals in the tables.

### Validation of Twitter percentiles using the fairness test

Bornmann et al. (2013a) proposed a statistical approach which can be used to study the ability of the TP to field-normalize TC (see also Kaur et al. 2013; Radicchi et al. 2008). The approach can be named as fairness test (Radicchi and Castellano 2012) and compares the impact results for the TP with that of bare TC with respect to field-normalization. We already used this test to study field-normalized Mendeley scores (Bornmann and Haunschild 2016).

In the first step of the fairness test (made for TP and TC separately), *all papers from 2012* are sorted in descending order by TP or TC, respectively. Then, the 10 % most frequently tweeted papers are identified and a new binary variable is generated, where 1 marks highly tweeted papers and 0 the rest.

In the second step of the test, all papers are grouped by the main disciplines as defined in the OECD field classification scheme.^3^ The OECD aggregates WoS subject categories (journal sets composed of Thomson Reuters) to the following broad fields: (1) natural sciences, (2) engineering and technology, (3) medical and health sciences, (4) agricultural sciences, (5) social sciences, and (6) humanities. Thomson Reuters assigns many journals to more than one WoS category. Thus, many papers in the TI correspondingly belong to more than one OECD field.

In the third step of the test, the proportion of papers belonging to the 10 % most frequently tweeted papers is calculated *for each OECD field*—using the binary variable from the first step. If TP were fair, the proportions within the fields should equal the expected value of 10 %. Furthermore, TC should show more and greater deviations from 10 % than TP.

The results of the fairness tests are shown in Table 3: TP is compared with TC. The table shows the total number of papers (published in TI journals) within the OECD fields and the proportion of papers within a field which belongs to the 10 % most frequently tweeted papers. Since there is no paper from the TI journals published in the humanities, this field could not be considered in the analyses.

**Table 3:** Number of papers and proportion of papers belonging to the 10 % most frequently tweeted papers in five main disciplines (as defined by the OECD)

OECD fields	Twitter counts (TC)		Twitter percentiles (TP)	
	Number of papers	Proportion top-10 %	Number of papers	Proportion top-10 %
Natural sciences	22,932	9.1	22,932	9.8
Engineering and technology	1838	**4.5**	1838	10.0
Medical and health sciences	29,192	8.9	29,192	10.2
Agricultural sciences	130	**0.8**	130	10.0
Social sciences	1603	**14.9**	1603	10.0
Humanities	0		0	

Some papers are counted more than once due to multiple field-assignment

The largest deviations of the proportions from 10 % can be expected for TC, because TC is not field-normalized. In the interpretation of the proportions in Table 3, we deem deviations of less than 2 % points acceptable (i.e. proportions with greater than 8 % and less than 12 %). The used bibliometric and Twitter data are erroneous, why we cannot expect exact results in Table 3. In other words, if the deviations of the proportions are within ±2 % around the expected value of 10 % (our range of tolerance), the normalization seems to be successful. We have printed in bold all proportions in the table with a deviation of more than ±2 %. As the results show, TC is outside the range of tolerance in three out of five fields. The social sciences reveal the largest deviation (with 14.9 %).

The TP shows the intended results in Table 3: All OECD fields have less than 1 % point deviations from 10 %; for three fields the proportion equals the expected value of 10 %. Thus, TC seems to field-normalize TC in all fields properly. However, following the argumentations of Sirtes (2012) and Waltman and van Eck (2013), the favorable results for TP could have a simple reason: The calculation of the TP is based on a field classification scheme which is also used for the fairness tests in Table 3 (the OECD aggregates journals, which we used for the normalization of TC). Therefore, Waltman and van Eck (2013) proposed to repeat the fairness test using another field categorization scheme: they used an algorithmically constructed classification system (ACCS). In 2012, Waltman and van Eck (2012) proposed the ACCS as an alternative scheme in bibliometrics to the frequently used field categorization scheme based on journal sets. The ACCS was developed using direct citation relations between publications. In contrast to the WoS category scheme, where a paper can be assigned to more than one field, each publication is assigned to only one field category in the ACCS. We downloaded the ACCS for the papers at the Leiden Ranking homepage^4^ and used it on the highest field-aggregation level (in order to compare the results with those based on the OECD fields).

The results of the comparison between TC and TP based on ACCS are shown in Table 4. In four out of five fields TC is outside the range of tolerance. The situation improves with percentile-based field normalization, but in mathematics and computer sciences as well in social sciences and humanities the proportions are significantly above the expected value of 10 %.

**Table 4:** Number of papers and proportion of papers belonging to the 10 % most frequently tweeted papers in five fields (as defined by the ACCS on the highest level)

Field	Twitter counts		Twitter percentiles	
	Number of papers	Proportion top-10 %	Number of papers	Proportion top-10 %
Biomedical and health sciences	26,940	11.5	26,940	10.0
Life and earth sciences	4145	**18.1**	4145	10.8
Mathematics and computer science	289	**19.0**	289	**13.1**
Physical sciences and engineering	10,565	**3.0**	10,565	9.6
Social sciences and humanities	2686	**20.7**	2686	**14.3**

Taken as a whole, the proportions for TC in Tables 3 and 4 reveal that field-normalization is generally necessary for tweets. Larger deviations from the expected value of 10 % are visible in most of the disciplines. However, the results of the study are ambivalent for TP: In natural sciences, life sciences, health sciences, and engineering TP seems to reflect field-normalized values, but in mathematics and computer science as well as in social sciences and humanities this does not seem to be the case.

### Comparison of countries based on Twitter percentiles

In the final part of this study, we use TP to rank the Twitter performance of countries in a first application of the new indicator. The analysis is based on all papers (from 2012) published by the countries which are considered in the TI. Since the results in the section “Validation of Twitter percentiles using the fairness test” show that the normalization of TC is only valid in biomedical and health sciences, life and earth sciences, mathematics and computer science, as well as physical sciences and engineering, we considered only these fields in the country comparison. These fields were selected on the base of the ACCS.

The Twitter impact for the countries is shown in Table 5. The table also presents the proportion of papers published by a country in the TI. As the results reveal, all proportions are less than 10 % and most of the proportions are less than 5 %. With a value of 8.1 %, the largest proportion of papers in the TI is available for the Netherlands. Thus, the calculation of the Twitter impact on the country level is generally based on a small proportion of papers. The tweets per paper vary between 16.9 (Denmark) and Taiwan (3.9). Both countries are also the most and less tweeted countries measured by TP (Denmark = 55.4, Taiwan = 45.6). The Spearman rank-order correlation between tweets per paper and TP is *r*_*s*_ = 0.9. Thus, the difference in both indicators to measure Twitter impact on the country level is small.

**Table 5:** Number of papers, number and proportion of papers in the Twitter Index, as well as sum of tweets, tweets per paper, and median Twitter percentiles for those countries with at least 1000 papers in the Twitter Index

Country	All papers	TI papers	Proportion of papers in TI	Sum of tweets	Tweets per paper	Median TP
Denmark	23,583	1330	5.6	22,460	16.9	55.4
Finland	20,502	1278	6.2	19,336	15.1	54.4
Norway	18,848	1009	5.4	12,349	12.2	53.1
UK	141,236	9956	7.0	143,125	14.4	52.1
Canada	98,649	6230	6.3	65,958	10.6	52.0
Australia	78,780	4787	6.1	54,173	11.3	51.5
Sweden	35,080	1947	5.6	27,052	13.9	51.2
Spain	90,510	3915	4.3	38,399	9.8	50.3
Belgium	28,780	1433	5.0	13,847	9.7	50.2
USA	724,091	50,823	7.0	686,221	13.5	50.1
Netherlands	60,390	4885	8.1	51,437	10.5	49.1
Switzerland	36,838	2379	6.5	24,495	10.3	49.0
Israel	18,467	1004	5.4	10,405	10.4	48.5
Brazil	82,866	1264	1.5	9050	7.2	48.4
Germany	168,769	8888	5.3	72,967	8.2	48.0
Japan	164,930	6137	3.7	46,473	7.6	48.0
Italy	124,321	4254	3.4	34,675	8.2	47.6
China	304,361	7225	2.4	37,071	5.1	47.5
India	76,138	1195	1.6	5603	4.7	47.5
France	138,431	6567	4.7	55,649	8.5	46.6
South Korea	97,786	2303	2.4	13,474	5.9	46.6
Taiwan	61,120	1776	2.9	6893	3.9	45.6

Many papers are multiply counted, because they belong to more than one country

## Discussion

While bibliometrics is widespread used to evaluate the performance of different entities in science, altmetrics offer a new form of impact measurement “whose meaning is barely understood” yet (Committee for Scientific and Technology Policy 2014, p. 3). The meaning of TC is especially unclear, because the meta-analysis of Bornmann (2015) shows that TC does not correlate with citation counts (but other altmetrics do). The missing correlation means for de Winter (2015) that “the scientific citation process acts relatively independently of the social dynamics on Twitter” (p. 1776) and it is not clear how TC can be interpreted. According to Zahedi et al. (2014) we thus need to study “for what purposes and why these platforms are exactly used by different scholars”. Despite the difficulties in the interpretation of TC, this indicator is already considered in the “Snowball Metrics Recipe Book” (Colledge 2014). This report contains definitions of indicators, which have been formulated by several universities—especially from the Anglo-American area. The universities have committed themselves to use the indicators in the defined way for evaluative purposes.

In this study, we have dealt with the normalization of TC. Since other studies have shown that there are field-specific differences of TC, the normalization seems necessary. However, we followed the recommendation of Bornmann (2014b) that TC should not be normalized on the level of subject categories, but a lower level (see here Zubiaga et al. 2014). We decided to use the journal level, since this level is also frequently used to normalize citations (Vinkler 2010). It is a further advantage of the normalization on the journal level that it levels out the practice of a substantial number of journals to launch a tweet for new papers in that journal: The practice leads to larger expected values for these journals. The problem with Twitter data is that many papers receive zero tweets or only one tweet. In order to restrict the impact analysis on only those journals producing a considerable Twitter impact, we defined the TI containing journals with at least 80 % tweeted papers. For all papers in each TI journal, we calculated TP which range from 0 (no impact) to 100 (highest impact). TP is proposed to use for cross-field comparisons.

We used the fairness test in order to study the field-independency of TP (in comparison with TC). Whereas one test based on the OECD fields shows favorable results for TP in all fields, the other test based on an ACCS points out that the TP can be validly used particularly in biomedical and health sciences, life and earth sciences, mathematics and computer science, as well as physical sciences and engineering. In a first application of TP, we calculated percentiles for countries whereby this analysis show that TP and TC are correlated on a much larger than typical level (*r*_*s*_ = 0.9). The high correlation coefficient points out that there are scarcely differences between the indicators to measure Twitter impact. The high correlation might be due to the fact that most of the papers used belong to only two fields (biomedical and health sciences and physical sciences and engineering) whereby the variance according to the fields is reduced between the papers.

This paper proposes a first attempt to normalize TC. Whereas Mendeley counts can be normalized in a similar manner as citation counts (Haunschild and Bornmann 2016), the low Twitter activity for most of the papers complicates the normalization of TC. In order to address the problem of low Twitter activity we defined the TI with the most tweeted journals. For 2012, the TI only contains 156 journals. However, we can expect that the journals in the TI will increase in further years, because Twitter activity will also increase. There is a high probability that the Twitter activity will especially increase in those fields where it is currently low (e.g. mathematics and computer science). The broadening of Twitter activities will also lead to a greater effectiveness of the percentile-based field-normalization, because the variance in fields will increase.

Besides further studies which address the normalization of TC and refine our attempt of normalization, we need studies which deal with the meaning of tweets. Up to now it is not clear what tweets really measure. Therefore, de Winter (2015) speculates the following: “It is of course possible that the number of tweets represents something else than academic impact, for example ‘hidden impact’ (i.e., academic impact that is not detected using citation counts), ‘social impact’, or relevance for practitioners … Furthermore, it is possible that tweets influence science in indirect ways, for example by steering the popularity of research topics, by faming and defaming individual scientists, or by facilitating open peer review” (de Winter 2015, p. 1776). When the meaning of TC is discussed, the difference between tweets and retweets should also be addressed. Retweets are simply repetitions of tweets and should actually be handled otherwise than tweets in an impact analysis (Bornmann and Haunschild 2015; Taylor 2013).

## Appendix

See Table 6.

**Table 6:** Journals in the Twitter Index (*n* = 156) sorted by the average tweets per paper published in 2012

Journal	Number of papers	Sum of tweets	Average tweets	Median number of tweets	Percentage of tweeted papers
*The Lancet*	239	11,246	47.1	21.0	100.0
*New England Journal of Medicine*	217	17,073	78.7	53.0	100.0
*Journal of Orthopaedic Trauma*	173	444	2.6	2.0	100.0
*British Dental Journal*	133	2129	16.0	12.0	100.0
*Cancer Cell*	114	797	7.0	6.0	100.0
*The Lancet Oncology*	107	1658	15.5	5.0	100.0
*Journal of Glaucoma*	101	222	2.2	2.0	100.0
*Age and Ageing*	147	734	5.0	3.0	99.3
*Science*	831	25,386	30.5	19.0	99.3
*BMC Medicine*	126	2048	16.3	8.0	99.2
*Cell Stem Cell*	122	1093	9.0	6.0	99.2
*Nature Climate Change*	120	3008	25.1	8.0	99.2
*Implementation Science*	119	1051	8.8	6.0	99.2
*PLoS Medicine*	115	3754	32.6	17.0	99.1
*Science Translational Medicine*	211	4995	23.7	16.0	99.1
*RNA*	201	716	3.6	3.0	99.0
*Journal of Medical Internet Research*	182	6398	35.2	19.0	98.9
*Pancreas*	174	249	1.4	1.0	98.9
*International Journal of Gynecological Cancer*	254	295	1.2	1.0	98.8
*British Medical Journal*	328	12,639	38.5	20.5	98.8
*Journal of the American Medical Association*	232	4382	18.9	8.5	98.7
*Journal of Pediatric Hematology/Oncology*	220	262	1.2	1.0	98.6
*Genome Biology*	140	1375	9.8	7.0	98.6
*Journal of Thoracic Oncology*	258	399	1.5	1.0	98.1
*Transplantation*	357	522	1.5	1.0	98.0
*International Journal of Public Health*	102	370	3.6	3.0	98.0
*Pediatric Emergency Care*	246	531	2.2	1.0	98.0
*Nature*	869	26,462	30.5	13.0	97.9
*Journal of Chemical Theory and Computation*	504	621	1.2	1.0	97.8
*Annals of Surgery*	301	1487	4.9	3.0	97.7
*Journal of Polymer Science Part B: Polymer Physics*	171	231	1.4	1.0	97.7
*BMC Medical Informatics and Decision Making*	159	1056	6.6	4.0	97.5
*Nature Geoscience*	148	1637	11.1	6.0	97.3
*Clinical Journal of Pain*	109	400	3.7	2.0	97.2
*Obesity Surgery*	246	625	2.5	2.0	97.2
*Archives of Internal Medicine*	103	2491	24.2	4.0	97.1
*Genome Research*	238	1990	8.4	6.0	97.1
*Nature Medicine*	164	2890	17.6	11.0	97.0
*Anesthesiology*	219	572	2.6	2.0	96.8
*Psychological Science*	213	7024	33.0	11.0	96.7
*Cell*	415	6337	15.3	7.0	96.4
*Genes, Brain and Behavior*	106	246	2.3	2.0	96.2
*Obesity Reviews*	101	929	9.2	5.0	96.0
*Nature Cell Biology*	126	594	4.7	3.0	96.0
*Journal of Nuclear Medicine*	242	451	1.9	2.0	95.9
*Journal of Burn Care and Rehabilitation*	142	200	1.4	1.0	95.8
*British Journal of Psychiatry, The*	117	1214	10.4	6.0	95.7
*BMC Infectious Diseases*	391	1157	3.0	2.0	95.7
*Journal of Cardiovascular Pharmacology*	138	153	1.1	1.0	95.7
*Microbes and Infection*	160	463	2.9	2.0	95.6
*PLoS Biology*	152	2799	18.4	7.0	95.4
*Journal of Spinal Disorders*	105	209	2.0	2.0	95.2
*Journal of the National Cancer Institute*	124	888	7.2	4.0	95.2
*Nature Genetics*	225	1963	8.7	6.0	95.1
*Clinical Nuclear Medicine*	118	258	2.2	2.0	94.9
*Organometallics*	998	1105	1.1	1.0	94.9
*Health Affairs*	268	5165	19.3	9.0	94.8
*Nature Methods*	150	974	6.5	4.0	94.7
*BMC Psychiatry*	236	1400	5.9	4.0	94.5
*Critical Care Medicine*	363	1427	3.9	2.0	94.5
*Nuclear Medicine Communications*	160	361	2.3	2.0	94.4
*Journal of Pediatric Gastroenterology and Nutrition*	283	754	2.7	2.0	94.3
*Inorganic Chemistry*	1560	1785	1.1	1.0	94.2
*Neurology*	389	3051	7.8	4.0	94.1
*Journal of Clinical Gastroenterology*	135	258	1.9	1.0	94.1
*Canadian Medical Association Journal*	114	1897	16.6	7.0	93.9
*Aging Cell*	112	359	3.2	3.0	93.8
*International Journal of Applied Earth Observation and Geoinformation*	140	178	1.3	1.0	93.6
*Macromolecular Bioscience*	166	175	1.1	1.0	93.4
*Immunity*	164	520	3.2	2.0	93.3
*BMC Neurology*	163	578	3.5	3.0	93.3
*Journal of Applied Ecology*	156	924	5.9	3.0	92.9
*Advanced Energy Materials*	169	184	1.1	1.0	92.9
*American Journal of Clinical Nutrition*	325	6421	19.8	6.0	92.6
*International Journal of Behavioral Nutrition and Physical Activity*	148	1372	9.3	5.0	92.6
*Scientific Reports*	794	12,934	16.3	3.0	91.9
*The American Journal of Human Genetics*	223	1163	5.2	3.0	91.9
*Journal of Occupational and Environmental Medicine*	195	369	1.9	1.0	91.3
*Pediatric Critical Care Medicine*	160	318	2.0	1.0	91.3
*Journal of the American Society for Mass Spectrometry*	228	242	1.1	1.0	91.2
*Neuro*-*Oncology*	159	282	1.8	1.0	91.2
*Journal of Clinical Investigation*	394	1543	3.9	2.0	91.1
*Journal of Nuclear Cardiology*	101	200	2.0	2.0	91.1
*Ecology Letters*	166	1054	6.3	4.0	91.0
*Macromolecular Rapid Communications*	261	254	1.0	1.0	90.8
*Cyberpsychology, Behavior, and Social Networking*	107	1359	12.7	3.0	90.7
*Macromolecular Chemistry and Physics*	265	260	1.0	1.0	90.6
*Science Signaling*	180	1432	8.0	7.0	90.6
*Nature Chemistry*	126	804	6.4	4.0	90.5
*Sexually Transmitted Infections*	125	655	5.2	4.0	90.4
*Nature Neuroscience*	225	2715	12.1	3.0	90.2
*Personality and Social Psychology Bulletin*	131	1970	15.0	5.0	90.1
*Steel Research International: A journal for steel and related materials*	131	125	1.0	1.0	90.1
*Medical Care*	179	353	2.0	1.0	89.9
*Nature Photonics*	118	448	3.8	2.0	89.8
*Journal of Animal Ecology*	134	350	2.6	2.0	89.6
*Dalton Transactions*	1709	1783	1.0	1.0	89.5
*British Journal of Sports Medicine*	198	3329	16.8	5.0	89.4
*Angewandte Chemie International Edition*	2227	4408	2.0	1.0	89.1
*World Journal of Surgical Oncology*	276	402	1.5	1.0	89.1
*The Oncologist*	180	612	3.4	3.0	88.3
*Nature Chemical Biology*	128	458	3.6	3.0	88.3
*BMC Public Health*	1125	5718	5.1	3.0	88.3
*Journal of Asthma*	152	635	4.2	3.0	88.2
*Medicine and Science in Sports and Exercise*	310	4307	13.9	3.0	88.1
*Nutrition Journal*	114	1497	13.1	5.0	87.7
*Free Radical Research*	137	138	1.0	1.0	87.6
*Chemistry Central Journal*	177	224	1.3	1.0	87.6
*Virology Journal*	320	679	2.1	2.0	87.5
*Shock*	183	220	1.2	1.0	87.4
*BMC Pregnancy and Childbirth*	164	865	5.3	3.0	87.2
*Pediatric Allergy and Immunology*	109	650	6.0	5.0	87.2
*American Journal of Clinical Oncology*	101	103	1.0	1.0	87.1
*Annals of Plastic Surgery*	250	264	1.1	1.0	86.8
*BMC Family Practice*	128	491	3.8	3.0	86.7
*Pain*	269	1001	3.7	2.0	86.6
*Pediatric Anesthesia*	170	316	1.9	2.0	86.5
*Journal of Clinical Psychology*	110	354	3.2	2.0	86.4
*Allergy*	194	1025	5.3	4.0	86.1
*Spine*	557	1852	3.3	1.0	86.0
*Critical Care* (*BMC*)	298	1041	3.5	2.0	85.9
*Journal of Strength and Conditioning Research*	390	3719	9.5	4.0	85.9
*Journal of Nutrition*	311	1089	3.5	2.0	85.9
*Journal of Experimental Medicine*	190	484	2.5	2.0	85.8
*European Journal of Neuroscience*	359	1050	2.9	2.0	85.5
*Journal of Hospital Medicine*	130	671	5.2	3.5	85.4
*Medical Education*	116	476	4.1	3.0	85.3
*Macromolecular Materials and Engineering*	116	107	0.9	1.0	85.3
*Psychosomatic Medicine*	122	333	2.7	1.0	85.2
*Clinical and Experimental Allergy*	169	722	4.3	4.0	85.2
*Acta Crystallographica Section C: Crystal Structure Communications*	229	224	1.0	1.0	84.3
*Nature Nanotechnology*	120	908	7.6	2.0	84.2
*Journal of Organometallic Chemistry*	410	362	0.9	1.0	84.1
*Obstetrics and Gynecology*	307	1749	5.7	2.0	84.0
*Anaesthesia*	149	413	2.8	2.0	83.9
*Journal of Feline Medicine and Surgery*	130	212	1.6	1.0	83.8
*Methods in Ecology and Evolution*	123	535	4.3	3.0	83.7
*Cell Reports*	242	839	3.5	2.0	83.5
*Journal of Child Psychology and Psychiatry and Allied Disciplines*	121	508	4.2	2.0	83.5
*Journal of Ecology*	139	390	2.8	2.0	83.5
*Anesthesia and Analgesia*	300	641	2.1	2.0	83.3
*Molecular Cell*	296	729	2.5	2.0	83.1
*Coordination Chemistry Reviews*	136	148	1.1	1.0	83.1
*Therapeutic Drug Monitoring*	103	209	2.0	1.0	82.5
*Journal of Clinical Psychopharmacology*	103	190	1.8	1.0	82.5
*Annals of Pharmacotherapy, The*	181	467	2.6	2.0	82.3
*Pediatrics*	698	8318	11.9	3.0	82.2
*Annals of Allergy, Asthma and Immunology*	145	684	4.7	3.0	82.1
*Brain*	280	1160	4.1	2.0	81.8
*Journal of the American Medical Informatics Association*	186	1058	5.7	2.0	81.7
*Conservation Biology*	114	420	3.7	2.0	81.6
*Nature Immunology*	124	319	2.6	2.0	81.5
*Plant Journal*	345	769	2.2	2.0	80.9
*American Journal of Psychiatry, The*	108	451	4.2	2.0	80.6
*Neuron*	340	2346	6.9	3.0	80.3
*Journal of Personality and Social Psychology*	141	1084	7.7	3.0	80.1

## Footnotes

1 http://www.altmetric.com.

2 http://feedback.impactstory.org/knowledgebase/articles/400281--highly-cited-and-other-impact-badges.

3 http://ipscience-help.thomsonreuters.com/incitesLive/globalComparisonsGroup/globalComparisons/subjAreaSchemesGroup/oecd.html.

4 http://www.leidenranking.com/methodology/fields.
